# Dosimetric considerations for moldable silicone composites used in radiotherapy applications

**DOI:** 10.1002/acm2.13605

**Published:** 2022-04-18

**Authors:** Ghada Aldosary, Jason Belec, Claire Foottit, Eric Vandervoort

**Affiliations:** ^1^ Department of Physics Carleton University Ottawa Ontario Canada; ^2^ Radiation Oncology Section Department of Oncology King Abdulaziz Medical City National Guard Health Affairs Riyadh Saudi Arabia; ^3^ Department of Medical Physics The Ottawa Hospital Cancer Centre Ottawa Ontario Canada; ^4^ Department of Medicine The University of Ottawa Ottawa Ontario Canada

**Keywords:** anthropomorphic, bolus, deformable, phantom, radiotherapy, silicone

## Abstract

Due to their many favorable characteristics, moldable silicone (MS) composites have gained popularity in medicine and recently, in radiotherapy applications. We investigate the dosimetric properties of silicones in radiotherapy beams and determine their suitability as water substitutes for constructing boluses and phantoms. Two types of silicones were assessed (ρ= 1.04 g/cm^3^ and ρ= 1.07 g/cm^3^). Various dosimetric properties were characterized, including the relative electron density, the relative mean mass energy‐absorption coefficient, and the relative mean mass restricted stopping power. Silicone slabs with thickness of 1.5 cm and 5.0 cm were molded to mimic a bolus setup and a phantom setup, respectively. Measurements were conducted for Co‐60 and 6 MV photon beams, and 6 MeV electron beams. The doses at 1.5 cm and 5.0 cm depths in MS were measured with solid water (SW) backscatter material (*D*_MS–SW_), and with a full MS setup (*D*_MS–MS_), then compared with doses at the same depths in a full SW setup (*D*_SW–SW_). Relative doses were reported as *D*_MS–SW_/*D*_MS–SW_ and *D*_MS–MS_/*D*_SW–SW_. Experimental results were verified using Monaco treatment planning system dose calculations and Monte Carlo EGSnrc simulations. Film measurements showed varying dose ratios according to MS and beam types. For photon beams, the bolus setup *D*_MS–SW_/*D*_SW–SW_ exhibited a 5% relative dose reduction. The dose for 6 MV beams was reduced by nearly 2% in a full MS setup. Up to 2% dose increase in both scenarios was observed for electron beams. Compared with dose in SW, an interface of MS–SW can cause relatively high differences. We conclude that it is important to characterize a particular silicone's properties in a given beam quality prior to clinical use. Because silicone compositions vary between manufacturers and differ from water/SW, accurate dosimetry using these materials requires consideration of the reported differences.

## 1 INTRODUCTION

There is a growing demand for solid materials that are moldable and water‐equivalent in radiotherapy, particularly for constructing patient‐customized bolus and deformable phantoms. As interest grows in adopting silicone composite materials for these applications, their suitability for dosimetric applications must be thoroughly understood.

Radiotherapy treatments are often prescribed with different types of bolus material to increase dose at the skin surface and/or compensate for missing tissue.^1^, ^2^, ^3^ There are several characteristics that bolus materials must fulfill,^4^ many of which can be met by certain moldable silicone (MS) composites.^5^ These materials can have similar mass densities as water's, and can also be manufactured to have similar tactile properties as human tissue's by modifying silicone formulations.

From a chemical point of view, silicones are generally categorized as synthetic polymers with a primary repeating unit of polydimethylsiloxane (PDMS). In addition to PDMS, silicones contain “filler” materials, which act to modify properties such as mechanical durability, hardness, and stickiness. Depending on the application and use, silicone is usually transformed into a stable composition through different chemical reactions. The details of these reactions can be found elsewhere,^6^ and the preferred reaction mechanism varies by application. To meet various application requirements, commercial silicones are also available with different formulations and instructions for curing. Due to practical reasons, such as ease of use, the favored mechanism for molding silicone materials used in medical applications^7^, ^8^ is platinum cure.^6^

Few experimental studies have investigated dose attenuation properties and tissue interface effects of silicone boluses in radiotherapy beams. Perhaps the first group to report on this were Dubois and Bice et al.^9^ They looked at two different forms of silicone and evaluated their use in 9 MeV electron beams. Compared with solid water (SW), they found that the dose reduction for these materials can be up to 52% at a depth of approximately 3 cm. More recently, and using improved silicone formulations, Canters et al.^10^ and Chiu et al.^11^ demonstrated the use of 3D printed molds to create patient‐specific boluses that offer superior contact with irregular patient surfaces with customizable shapes and thicknesses compared with standard synthetic gel‐slab bolus. For 6 MeV and 9 MeV beams, Chiu et al.^11^ reported that in vivo measurements made with platinum cure silicone bolus were within 5% of the prescribed dose.

In addition to the use of silicone composites as bolus, there has been recent interest in employing these materials for constructing radiotherapy anthropomorphic phantoms. For example, the durability and flexibility of these materials make them useful for constructing deformable phantoms for adaptive radiotherapy and magnetic resonance guided radiotherapy. Applications include deformable phantoms for various anatomical sites, such as the thorax,^12^ prostate,^13^, ^14^ liver,^15^ and breast.^5^ In these studies, dose measurements were conducted using radiochromic film,^13^, ^15^ optically simulated luminescent dosimeters,^13^ ionization chambers (ICs),^5^ or scintillators^15^; however, a thorough investigation of the dosimetric properties of silicone has yet to be reported.

The purpose of this work is to investigate the dosimetric properties of MS composites in high‐energy photon and electron beams, and to determine their suitability as water substitutes for constructing bolus materials and radiotherapy phantoms.

## 2 MATERIALS AND METHODS

In this study, we investigated two types of two‐part composite platinum cure MSs using experimental measurements, treatment planning system (TPS) calculations, and Monte Carlo (MC) simulations. Specifically, we sought to answer two questions: the first is, are there differences in high‐energy photon and electron radiation beam absorptions in MS compared with SW? And the second is, how do these differences change when an interface of MS and SW is introduced at different depths? These questions are relevant to consider for bolus and deformable phantom construction. For bolus, the dose at the interface between the silicone material and skin is of concern to clinical dose prescription. For deformable phantom construction, it may be desirable to fix a dosimeter rigidly in SW within a surrounding deformable media to reduce measurement uncertainty.

Ecoflex^™^ 00–10 (E10) and Ecoflex 00–50^™^ (E50) (Smooth‐On Inc., PA, USA) MS were used. They are described by the manufacturer as white‐translucent silicone rubbers. They both have a low viscosity, and are soft, yet durable, and were selected to characterize the extreme ends of this product line's range. These materials are reported to stretch to many times their original size without tearing and return to their original form without distortion. This is supported by the mechanical properties listed in Table 1.

**TABLE 1:** Physical and mechanical properties of commercial moldable silicones used in this study, as reported by the manufacturer (Smooth‐On, Inc., PA, USA)

Manufacturer reported physical property	Ecoflex^™^ 00–10 (E10)	Ecoflex^™^ 00–50 (E50)
Mass density (g/cm^3^)	1.04	1.07
Shore hardness	00–10	00–50
100% Modulus (kPa)	55.2	83.0
Tensile strength (kPa)	827.4	2171.8

For any material of interest, it is possible to use stoichiometric data to determine key theoretical physical quantities that are relevant for evaluating radiation absorption of materials, such as the mass density (ρ), relative electron density (RED) (*Z*_eff_), mean excitation energy, relative mean mass energy‐absorption coefficient ratios ${\left(\bar{\frac{\mu }{\rho }}\right)}_{\mathrm{water}}^{\mathrm{med}}$, and the relative mean mass restricted stopping power ratios for a medium$\;{\left(\bar{\frac{L}{\rho }}\right)}_{\mathrm{water}}^{\mathrm{med}}$. Because the exact formulation of E10 and E50 are considered proprietary information and were not made available by the manufacturer, the formula for generic silicone^6^ was used to determine silicone's quantities (i.e., C_2_H_6_OSi), and filler material was not quantified. This assumption was also based on the fact that filler material is usually added in small amounts as parts per million. Table 2 lists stoichiometric data for this generic form of silicone, SW, and water that were used to determine the aforementioned quantities. In this work, the effective atomic number (*Z*_eff_) values were calculated using the classic Mayneord formula.^16^ The mean excitation energy was obtained from NIST's ESTAR database.^17^ The RED, mean mass energy‐absorption coefficient ratios, ${\left(\bar{\frac{\mu }{\rho }}\right)}_{\mathrm{water}}^{\mathrm{med}}$, for Co‐60 and 6 MV spectra, as well as the mean restricted stopping power ratios,$\;{\left(\bar{\frac{L}{\rho }}\right)}_{\mathrm{water}}^{\mathrm{MS}}($with a cut‐off energy of Δ=10keV for Co‐60 and 6 MV spectra) were determined using the same method reported by Ho and Paliwal^18^ and Cunningham and Schulz,^19^ and by using data from the NIST ESTAR^17^ and XCOM^20^ databases.

**TABLE 2:** Stoichiometric data and fractional weight of each element found in different media of interest used in this study. Each element is listed with its atomic number, *Z*, provided in brackets

	Fractional weight						
Medium	H (1)	C (6)	N (7)	O (8)	Si (14)	Cl (17)	Ca (20)
Silicone	0.081	0.324	–	0.216	0.379	–	–
Solid Water (RMI457)	0.081	0.672	0.024	0.198	–	0.001	0.023
Water	0.112	–	–	0.888	–	–	–

### 2.1 Description of phantoms

**FIGURE 1:**
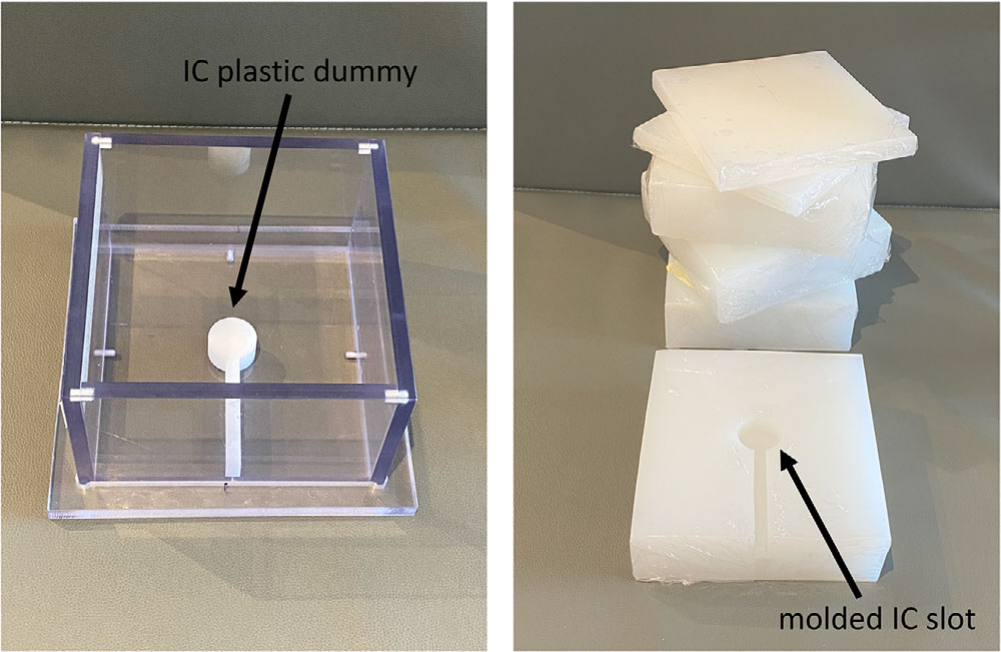
The molding process for the silicone slabs included using a custom‐built acrylic open faced cuboid container, which had an optional Markus IC dummy insert that can be added at the base to form a slot for IC placement. The molded E10 and E50 silicone slabs are shown on the right‐hand image, and were 1.5 cm and 5.0 cm thick

A custom‐built, acrylic cuboid (with a 15 × 15 cm^2^ inner base area, 6 mm wall thickness, 10 cm height) was used as a mold for constructing silicone slabs with variable thicknesses. This allowed measurements to be performed in a simple, reproducible geometry. Six silicone slabs were constructed: three using E10, and three using E50—each three corresponding to each silicone type having different thicknesses. The first slab types were 1.5 cm thick with a 15 × 15 cm^2^ base area. The second were 5.0 cm thick with a 15 × 15 cm^2^ base area. The third was also 5.0 cm thick with a 15 × 15 cm^2^ base area, and had an enclosed embedded slot for securely positioning an Advanced Markus® plane–parallel IC (S/N: 00815, Model TN34045, PTW Freiburg, Germany) flush against one of the slab's surfaces. The slot was created by placing a plastic IC dummy, with exact dimensions as the Markus IC, at the central axis on the base of the mold to create a slot for positioning the IC. Figure 1 shows the custom‐built mold, Markus IC dummy, and molded silicone slabs. Both types of silicone were left to cure for a minimum of 4 h. Since silicone is a deformable material, the total uncertainties related to producing and setting up silicone slabs with the stated thicknesses were determined by measuring the dimensions of cured silicone slabs with a caliper (within 0.1% measurement precision).

In addition to the six silicone phantom slabs, four Solid Water^®^ slabs (Gammex‐RMI, WI, USA) were used in our experimental measurements. One of these slabs was 1.5 cm thick, two were 5.0 cm thick, and the fourth was also 5.0 cm thick with an embedded slot to fit the Markus IC flush against one of its surfaces.

### 2.2 Experimental setup

**FIGURE 2:**
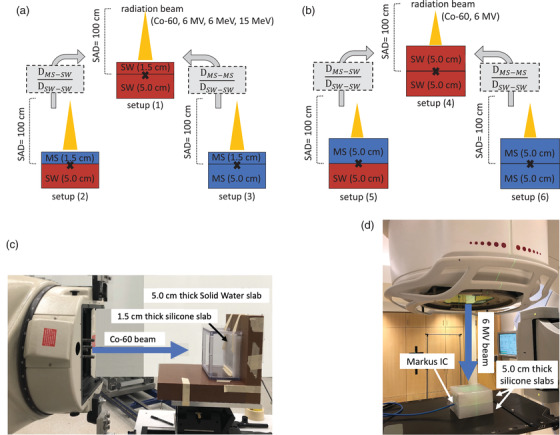
Pictoral representations of the experimental setups used for each beam type. Photon beams were measured at depths of 1.5 cm and 5.0 cm: setups (1)–(6) as shown in (a) and (b). Electron beams were measured using 1.5 cm depth slabs: setups (1), (2), and (3) as shown in (a). IC measurements were conducted in the lower MS slabs that were made to fit the IC flush against its surface. The measurement points (at the interfaces) are identified with the x marker in the illustrations shown in (a) and (b) and evaluated dose ratios are shown in grey boxes. Measurements are compared to Monaco TPS calculations for 6 MV and to EGSnrc Monte Carlo simulations for Co‐60 and 6 MV at the same depths. An example of one of the setups used for measurements in the Co‐60 beam is provided in (c), in which the sides of the acrylic mold were used as a frame to maintain the silicone slabs in an upright position for a lateral beam orientation. An example of one of the setups used for measurements in the 6 MV beam, using the Markus IC, is provided in (d). Measurements for 6 MeV beams were conducted with a 10 × 10 cm^2^ electron applicator in a similar setup to that shown in (d)

This study focused on two main aspects of using silicone; when it is used as a full medium for absorbed dose measurements, or when a certain thickness of silicone is placed on top of another type of medium, creating an interface at the point of dose measurement. Figure 2 provides a pictorial representation of the six slab configurations used experimentally in computed tomography (CT) imaging, TPS calculations, and in MC simulations. In this work, all Co‐60 photon measurements were performed using a primary standard Co‐60 gamma teletherapy irradiator (GammaBeam X200™, Best Theratronics Ltd., Ottawa, Ontario, Canada). All 6 MV photon measurements were performed using a clinical linear accelerator (Elekta Synergy, Elekta Instrument AB, Stockholm, Sweden). And all 6 MeV electron measurements were performed using another clinical linear accelerator (Elekta Infinity, Elekta Instrument AB, Stockholm, Sweden). Details of each process are provided separately below for each beam type and energy, and for radiochromic film and IC measurements.

### 2.3 Radiochromic film

Radiochromic film is known to have negligible effects on radiation fluence,^21^ therefore, EBT3 Gafchromic (Ashland Inc., Wayne, NJ, USA) film measurements were also performed to validate IC measurements acquired at phantom slab interfaces. Films were pre‐cut and divided into two pieces. For each irradiation, a larger piece (10.16 cm × 10.16 cm) was used for film dose measurement, and a smaller piece (2.54 cm x 10.16 cm) was used as dedicated control piece to account for darkening due to heat and light exposure and to estimate unirradiated film baseline homogeneity and scan repeatability. Because two separate batches of films were used for Co‐60 measurements and for linac measurements, two separate calibrations were performed. For Co‐60 measurements, film calibration was performed using the Co‐60 irradiator. At the time of measurements, it had a nominal dose rate of 48.8 cGy/min at a reference depth of 5.0 cm in water, for a 10 × 10 cm^2^ field size, 100 cm source‐to‐surface distance (SSD). For linac measurements, film calibration was performed using the 6 MV beam. Film calibration was performed following a procedure similar to what was described by Devic et al.^22^ Film orientation was maintained by marking the upper left edge of the film, and by using a custom‐made template which exactly fits both the measurement film as well as its control piece. The films were always placed in the same location on the scanner bed for pre‐irradiation and post‐irradiation scans. Each film was scanned three times then averaged, and three warm‐up scans were taken prior to scanning. All films were scanned and irradiated using the same configuration: in transmission mode, 48‐bit color, 150 DPI, with an Epson 10000XL scanner (Seiko Epson Corporation, Suwa, Nagano, Japan), and by using the red channel for Co‐60 measurements, and the green channel for linac measurements. Film readout was performed using MATLAB (MathWorks, Inc., Natick, MA, USA, v. R2020b) and by using a 0.3 cm radius region‐of‐interest sampled to the center of each film. An average of the mean net optical density in each region of interest was used to calculate the average dose for each setup.

### 2.4 Measurements in photon and electron beams

For each of the configurations shown in Figure 2a,b, dose measurements were performed using film and the Markus IC at the central‐axis position. Measurements in all beam types were conducted with the IC protection cap on, then repeated for 6 MV and 6 MeV beams with the IC protection cap off to distinguish chamber cap‐related perturbation effects in measurement data. IC measurements were conducted using a Keithley electrometer (S/N: 8–8278, Model 35040, Advanced Therapy Dosimeter, Fluke Biomedical, Everett, WA, USA) set on 300 V bias. Co‐60 measurements were performed at depths of 1.5 cm and 5.0 cm, 10 × 10 cm^2^ field size, 100 cm source‐to‐axis distance (SAD) and using an irradiation time of 2.05 min to deliver 100.0 cGy at the measurement point. 6 MV photon beam measurements were performed at depths of 1.5 cm and depths of 5.0 cm, 10 × 10 cm^2^ field size, 100 cm SAD, and 1000 Monitor Units (MU, see setup in Figure 2a,b). 6 MeV beam measurements were performed at a depth of 1.5 cm, 10  ×  10 cm^2^ electron applicator size, 100 cm SAD, and 1000 MU. The primary standard Co‐60 beam utilized a fixed gantry head geometry to irradiate with a highly precise and reproducible lateral beam setup. Because the silicone slabs could sag when positioned on their short side, the acrylic mold was used as a frame to maintain the silicone slabs in a flat upright position for a lateral beam orientation, as shown in Figure 2c. This kept the beam direction orthogonal to the slab surface. The base of the mold was removed so it would not interfere with the dose readings. The low‐dose rate of the Co‐60 beam required long irradiation times to achieve a sufficient dose level. Due to this reason, and because of the slab design, the lateral irradiation geometry, and limited access to the Co‐60 irradiator, only one film was exposed per experimental setup. This limited access also prevented repeating IC measurements with the protection cap off. Measurements conducted using the linac were performed using a vertical beam orientation (see Figure 2d). In order to reduce the overall uncertainty on film readings^23^ for these experiments, four pieces of film were stacked on top of each other and irradiated simultaneously.

Three dose readings were obtained for each beam type, beam energy, and setup configuration. Then, dose ratios were determined as illustrated in Figures 2a,b, such that the dose measured with an upper slab of silicone and a lower slab of solid water (MS–SW) was normalized to the dose measured with an upper slab of solid water and a lower slab of solid water (SW–SW): that is, $D_{\mathrm{SW}-\mathrm{SW}}^{\mathrm{MS}-\mathrm{SW}}$ and $D_{\mathrm{SW}-\mathrm{SW}}^{\mathrm{MS}-\mathrm{SW}}$. Similarly, an average of the three readings was used to determine the dose measured with an upper slab of silicone and a lower slab of silicone (MS–MS), relative to the dose measured with an upper slab of solid water and a lower slab of solid water (SW–SW): i.e., $D_{\mathrm{SW}-\mathrm{SW}}^{\mathrm{MS}-\mathrm{MS}}$ and $D_{\mathrm{SW}-\mathrm{SW}}^{\mathrm{MS}-\mathrm{MS}}$. Alongside these ratios, the total uncertainties for film and IC dose measurements were estimated by considering the film calibration process, the precision of the silicone molding process, dose calibration factors, beam setup, silicone slab thickness variability due to sag during measurements, as well as setup uncertainty and dose/ reading reproducibility, where applicable.

$D_{\mathrm{SW}-\mathrm{SW}}^{\mathrm{MS}-\mathrm{SW}}$ and $D_{\mathrm{SW}-\mathrm{SW}}^{\mathrm{MS}-\mathrm{MS}}$ values and associated uncertainties were then compared to values determined from TPS calculations and MC simulations, as described below.

### 2.5 CT imaging and TPS calculations

Using the slab orientation for vertical beam irradiation (Figure 2d), CT images of the six configurations shown in Figure 2a,b were acquired with a radiotherapy CT simulator (Brilliance Big Bore, Philips Medical Systems, Cleveland, USA), and with an image resolution of 0.4 mm  ×  0.4 mm × 0.4 mm, 120 kVp, and 350 mAs. In each setup, four pieces of film were placed between the two slabs. The image sets were imported into the Monaco^®^ TPS (v. 5.11.02, Elekta Instrument AB, Stockholm, Sweden), the external contours of each slab were contoured, and a small region of interest (0.3 cm^3^ volume) centrally located in film was also contoured.

The Monaco^®^ TPS^24^ uses a specified CT‐to‐electron density (ED) table to convert a CT image pixel's Hounsfield Unit (HU) value to an ED value.^25^ The HU values for each image pixel in the contoured structure are mapped to RED values using a user specified CT‐to‐ED file. This file is based on measurement data obtained with a phantom, such as the Gammex 467 Tissue Characterization Phantom (Gammex Inc., Middleton, WI, USA). These type of phantoms house inserts made of tissue‐equivalent materials with standard compositions^26^ such as lung, adipose, water, muscle, cartilage, bone, aluminum, and iron. Once the ED is determined, this value is subsequently used by the dose calculation algorithm to determine material characteristics required for dose calculation such as, mass density (ρ), photon mass attenuation coefficient ($\frac{\mu }{\rho }$), electronic (collisional) mass collisional stopping power ($\frac{S_{\mathrm{col}}}{\rho })$, electron scattering power, etc. Consequently, for accurate dose calculation using the TPS, it is important to use the correct RED value for a particular material.

When plastic or silicone materials are used, the CT‐to‐ED file may not be appropriate to apply directly since the compositions of these materials can differ from tissues’. To ensure that TPS dose calculations were free of systematic errors resulting from a potential material misrepresentation, the correct RED value of 0.983 (see Table 3) was applied by overriding the silicone slab contours’ voxels during calculations. The RED value automatically reported by the TPS was measured at the center of each silicone slab and noted for comparison purposes only.

**TABLE 3:** Physical quantities related to radiation attenuation and absorption, as reported for generic silicone, and compared to common materials used in radiotherapy dosimetry (namely, solid water and water)

	Material		
Physical quantity	Silicone	Solid Water (RMI457)	Water
ρ (g/cm^3^)	1.01	1.03	1.00
*Z*_eff_	10.65	7.40	7.42
RED	0.983	1.01	1.00
Mean excitation energy (eV)	93.80	70.00	75.00
${\left(\bar{\frac{\mu }{\rho }}\right)}_{\mathrm{water}}^{\mathrm{med}}$ for Co‐60	0.975	0.944	1.00
${\left(\bar{\frac{\mu }{\rho }}\right)}_{\mathrm{water}}^{\mathrm{med}}$ for 6 MV	0.975	0.973	1.00
$\;{\left(\bar{\frac{L}{\rho }}\right)}_{\mathrm{water}}^{\mathrm{med}}$ for Co‐60, with Δ=10keV	0.930	1.067	1.00
$\;{\left(\bar{\frac{L}{\rho }}\right)}_{\mathrm{water}}^{\mathrm{med}}$ for 6 MV, with Δ=10keV	0.929	1.080	1.00

A treatment plan was generated for each of the setup configurations in accordance with measurement conditions. The plan isocentre (100 cm SAD) was set as the interface of the two slabs. For 6 MV photon beams, both 1.5 cm thick and 5.0 cm thick upper slabs were used. For 6 MeV electron beams, only 1.5 cm thick upper slabs were used. This is because 5 cm of material is beyond the practical range of 6 MeV electron beams.

Two dose calculation algorithms were employed for 6 MV photon beam calculations, Collapsed Cone Convolution (CC) and X‐ray Voxel Monte Carlo (XVMC)^27^ MC implementation. The dose‐to‐medium was calculated using 0.2 cm grid spacing, and for MC with 0.1 uncertainty. The mean dose for the small film contour was obtained for each plan. The beam model used for all calculations was for an Agility MLC linear accelerator (Elekta, Instrument AB, Stockholm, Sweden). For 6 MeV, the VMC^28^ MC implementation dose calculation algorithm was used to calculate dose‐to‐medium with 0.2 cm grid spacing, and 10^6^ histories, and similar to 6 MV plans, the mean dose for the small film contour was also obtained for each plan. In accordance with experiments, for 6 MV and 6 MeV, the values reported from TPS calculations are $D_{\mathrm{SW}-\mathrm{SW}}^{\mathrm{MS}-\mathrm{SW}}$ and $D_{\mathrm{SW}-\mathrm{SW}}^{\mathrm{MS}-\mathrm{MS}}$ for depths of 1.5 cm (for photon and electron plans) and 5.0 cm (for photon plans).

### 2.6 Monte Carlo simulations

In order to validate experimental data and TPS calculations with photon beams, MC simulations were carried out using EGSnrc/DOSXYZnrc.^29^, ^30^ Voxelized dose calculation geometry files were created to emulate the experimental setups and phantom material geometries described above. MC simulation properties are summarized in Table 4, as recommended by AAPM's Research Committee Task Group 268.^31^ For each energy, and all the configurations shown in Figure 2a,b, the dose was scored for a 1.0 × 1.0 cm^2^ region‐of‐interest area and sampled along the central‐axis of the beams in phantom material at 100.0 cm SAD ± 0.5 cm in 0.1 cm increments. For each energy and depth, the dose was then normalized to the dose obtained at 100 cm SAD for a SW–SW setup (i.e., $D_{\mathrm{SW}-\mathrm{SW}}^{\mathrm{MS}-\mathrm{SW}}$ and $D_{\mathrm{SW}-\mathrm{SW}}^{\mathrm{MS}-\mathrm{MS}}$).

**TABLE 4:** Summary of simulation properties and parameters used to simulate radiation dose attenuation in silicone and solid water phantoms

Item name	Description	References
System/code, version/release date	EGSnrc code system/DOSXYZnrc, v2021/ 2000	Kawrakow et al. [29]
Validation	Simulation results are compared with experimental measurements	See Figure 2 for diagram of experimental conditions
Timing	Simulation times ranged from 2.092 to 3.517 single CPU hours (2.7 GHz)	–
Source Description	HEN_HOUSE input spectra data files: co60.spectrum (Co‐60) and mohan6.spectrum (6 MV)	Kawrakow et al. [30]
Cross‐section data	PEGLESS –mode Using silicone and Solid Water stoichiometric data	Kawrakow *et al*. [30] See *Tables* *2* and *3*
Transport parameters	Boundary crossing algorithm: EXACT Electron‐step algorithm: PRESTA‐II Photon cut‐off energy: 1 keV Electron cut‐off energy: 512 keV Spin effects: On Bremmstrahlung angular sampling: simple Bethe–Heitler Bremssrahlung cross‐sections: On	–
Variance reduction techniques	None	–
Scored quantities	Absorbed dose to medium	–
# Histories/statistical uncertainty	7 × 10^9^/ ≤0.3% (*k *= 1)	–
Statistical methods	History‐by‐history	Kawrakow et al. [30]
Postprocessing	None	–

## 3 RESULTS

Table 3 listed the dosimetric quantities determined for generic silicone in comparison with SW and water. While the mass density of all three materials is similar, quantities such as *Z*_eff_, the RED, the mean excitation energy, and ${\left(\bar{\frac{L}{\rho }}\right)}_{\mathrm{water}}^{\mathrm{med}}$ are significantly different for silicone. Within the energy range investigated, ${\left(\bar{\frac{\mu }{\rho }}\right)}_{\mathrm{water}}^{\mathrm{med}}$is predominantly due to incoherent scattering (Compton interactions).

The combined uncertainties (*k *= 1) for measurements conducted in all beam types and energies were 0.92% for IC in all beam types and energies (refer to Table 5), and for film were 2.06% for Co‐60, and 1.11% for 6 MV and 6 MeV (refer to Table 6). In Tables 5 and 6, Type A uncertainties are evaluated through statistical analysis of measurements (such as standard deviation and standard error around the mean of results), whereas Type B uncertainties are determined through best scientific judgment based on the literature.^23^, ^32^, ^33^

**TABLE 5:** Uncertainty budget for dose value readings obtained with the Markus ionization chamber in both photon (Co‐60 and 6 MV) and 6 MeV electron beams

Category of uncertainty	Source of uncertainty	Uncertainty (%)	Evaluation Type (A or B)	Remark
Measurement Setup	Front‐pointer setting	0.03	A	Measured
	Field size setting	0.02	A	Measured
	Depth setting (drilling accuracy)	0.17	A	Measured
	Temperature and pressure variation	0.01	A	Measured
	Humidity change	0.05	A	Measured
	Silicone slab thickness variation (sag)	0.26	A	Measured
	Shutter error (for Co‐60 beams)	0.00	A	Measured shutter error is 3 ms
Ionization chamber‐related	$N_{D,W}^{Co-60}$	0.50	A	Obtained directly at the standard lab
	Ionization chamber stability	0.00	A	Measured
	Leakage current	0.05	A	Measured
	Solid water phantom material variability	0.70	B	Source: AAPM TG‐51 Addendum [33]
*Combined uncertainty (k = 1)*		0.92		
*Combined uncertainty (k = 2)*		1.84		

**TABLE 6:** Uncertainty budget for net optical density readings obtained with EBT3 film in both photon (Co‐60 and 6 MV) and 6 MeV electron beams

		Uncertainty (%)			
Category of uncertainty	Source of uncertainty	Co‐60 beam	6 MV and 6 MeV beams	Evaluation Type (A or B)	Remark
Measurement setup	Front‐pointer setting	0.03	0.03	A	Measured
	Field size setting	0.02	0.02	A	Measured
	Depth setting (drilling accuracy)	0.17	0.17	A	Measured
	Temperature and pressure variation	0.01	0.00	A	Measured
	Humidity change	0.05	0.00	A	Measured
	Silicone slab thickness variation (sag)	0.26	0.26	A	Measured
	Shutter error (for Co‐60 beams)	0.00	0.00	A	Measured shutter error is 3 ms
Ionization chamber‐related	$N_{D,W}^{Co-60}$	0.50	0.00	A	Obtained directly at the primary standard lab
	Ionization chamber stability	0.00	0.00	A	Measured
	Leakage current	0.05	0.05	A	Measured
	Solid water phantom material variability	0.70	0.70	B	Source: AAPM TG‐51 Addendum [33]
EBT3 Film‐related	Scanner uniformity	0.28	0.28	B	Source: Van Battum et al. [23]
	Lateral correction	1.00	0.00	A	Measured
	Calibration curve fitting	0.50	0.30	A	Measured
	Intra‐batch variations	0.28	0.28	A	Measured
	Background	0.50	0.00	A	Measured
	Energy dependence	0.50	0.00	B	Source: Van Battum et al. [23]
	Angular dependence	0.50	0.00	B	Source: Van Battum et al. [23]
	Intrinsic film homogeneity	1.10	0.60	B	Source: Van Battum et al. [23]
*Combined uncertainty (k = 1)*		2.06	1.11		
*Combined uncertainty (k = 2)*		4.12			

Table 7 provides a comparison of $D_{\mathrm{SW}-\mathrm{SW}}^{\mathrm{MS}-\mathrm{SW}}$ and $D_{\mathrm{SW}-\mathrm{SW}}^{\mathrm{MS}-\mathrm{MS}}$ values in phantom material at the measurement plane from experimental measurements and MC simulations in the Co‐60 photon beam. In addition to experimental measurements and MC simulation ratios, the ratios obtained from TPS calculations are also listed for the 6 MV photon beam and 6 MeV electron beams in Tables 8 and 9, respectively. In Tables 7, 8, 9, experimental data for the two silicone types are provided separately, whereas data from TPS calculations and MC simulations are provided for the generic form of silicone. MC (DOSXYZnrc) data closely matched those obtained experimentally with film at the same depth.

**TABLE 7:** $D_{\mathrm{SW}-\mathrm{SW}}^{\mathrm{MS}-\mathrm{SW}}$ and $D_{\mathrm{SW}-\mathrm{SW}}^{\mathrm{MS}-\mathrm{MS}}$ values at 100 SAD and variable depths, in a Co‐60 photon beam from experimental measurements and MC simulations (DOSXYZnrc). Note that MC simulations were performed for a generic form of silicone, therefore the same simulation output data is provided for both types of silicone (E10 and E50)

		$D_{\mathrm{SW}-\mathrm{SW}}^{\mathrm{MS}-\mathrm{SW}}$		$D_{\mathrm{SW}-\mathrm{SW}}^{\mathrm{MS}-\mathrm{MS}}$	
Depth (cm)	Method	E10 Silicone	E50 Silicone	E10 Silicone	E50 Silicone
***1.5***	*Markus IC (cap on)*	0.943 ± 0.013	0.945 ± 0.013	0.951 ± 0.013	0.945 ± 0.013
	*Film*	0.950 ± 0.028	0.959 ± 0.028	1.009 ± 0.029	1.025 ± 0.030
	*MC (DOSXYZnrc)*	0.983 ± 0.004		0.990 ± 0.004	
***5.0***	*Markus IC (cap on)*	0.937 ± 0.013	0.935 ± 0.013	0.925 ± 0.013	0.932 ± 0.013
	*Film*	0.968 ± 0.028	0.983 ± 0.029	1.037 ± 0.030	1.018 ± 0.030
	*MC (DOSXYZnrc)*	0.991 ± 0.004		1.001 ± 0.004	

**TABLE 8:** $D_{\mathrm{SW}-\mathrm{SW}}^{\mathrm{MS}-\mathrm{SW}}$ and $D_{\mathrm{SW}-\mathrm{SW}}^{\mathrm{MS}-\mathrm{MS}}$ values at 100 cm SAD and two depths, in a 6 MV photon beam from experimental measurements, TPS‐CC calculations (using collapsed cone convolution algorithm), TPS‐MC (using Monaco's MC calculation algorithm) and MC simulations (DOSXYZnrc). Note that MC simulations and TPS calculations were performed for a generic form of silicone, therefore the same resulting output data are provided for both types of silicone (E10 and E50)

		$D_{\mathrm{SW}-\mathrm{SW}}^{\mathrm{MS}-\mathrm{SW}}$		$D_{\mathrm{SW}-\mathrm{SW}}^{\mathrm{MS}-\mathrm{MS}}$	
Depth (cm)	Method	E10 Silicone	E50 Silicone	E10 Silicone	E50 Silicone
***1.5***	*Markus IC (cap on)*	0.960 ± 0.012	0.961 ± 0.012	0.957 ± 0.012	0.953 ± 0.012
	*Markus IC (cap off)*	0.957 ± 0.012	0.964 ± 0.013	0.960 ± 0.013	0.957 ± 0.012
	*Film*	0.970 ± 0.012	0.959 ± 0.012	0.998 ± 0.012	0.980 ± 0.012
	*TPS‐CC*	1.001 ± 0.011		0.998 ± 0.011	
	*TPS‐MC*	1.001 ± 0.008		0.999 ± 0.013	
	*MC (DOSXYZnrc)*	0.979 ± 0.003		1.009 ± 0.003	
***5.0***	*Markus IC (cap on)*	0.932 ± 0.012	0.947 ± 0.012	0.944 ± 0.012	0.939 ± 0.012
	*Markus IC (cap off)*	0.934 ± 0.012	0.954 ± 0.012	0.951 ± 0.012	0.948 ± 0.012
	*Film*	0.948 ± 0.011	0.960 ± 0.012	0.995 ± 0.012	0.982 ± 0.012
	*TPS‐CC*	0.992 ± 0.018		0.996 ± 0.017	
	*TPS‐MC*	0.998 ± 0.018		0.996 ± 0.017	
	*MC (DOSXYZnrc)*	0.978 ± 0.003		1.009 ± 0.003	

**TABLE 9:** $D_{\mathrm{SW}-\mathrm{SW}}^{\mathrm{MS}-\mathrm{SW}}$ and $D_{\mathrm{SW}-\mathrm{SW}}^{\mathrm{MS}-\mathrm{MS}}$ values at 100 cm SAD and 1.5 cm depth, in a 6 MeV electron beams from experimental measurements and TPS‐MC (using Monaco's MC Calculation Algorithm). Note that TPS calculations were performed for a generic form of silicone, therefore the same calculation data are provided for both types of silicone (E10 and E50)

		$D_{\mathrm{SW}-\mathrm{SW}}^{\mathrm{MS}-\mathrm{SW}}$		$D_{\mathrm{SW}-\mathrm{SW}}^{\mathrm{MS}-\mathrm{MS}}$	
Depth (cm)	Method	E10 Silicone	E50 Silicone	E10 Silicone	E50 Silicone
***1.5***	*Markus IC (cap on)*	1.022 ± 0.013	1.001 ± 0.013	1.027 ± 0.013	1.000 ± 0.013
	*Markus IC (cap off)*	1.012 ± 0.013	0.992 ± 0.013	1.013 ± 0.013	0.988 ± 0.013
	*Film*	1.003 ± 0.012	0.983 ± 0.012	1.041 ± 0.012	1.019 ± 0.012
	*TPS‐MC*	1.005 ± 0.005		0.988 ± 0.005	

**FIGURE 3:**
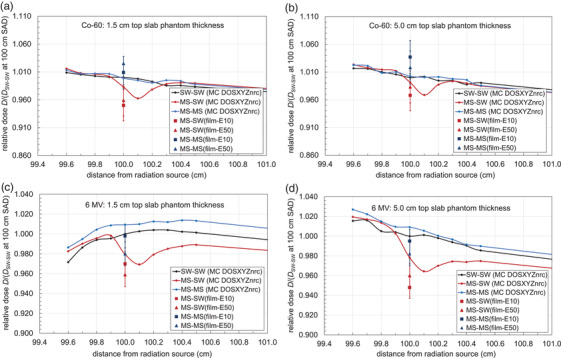
Monte Carlo (MC, DOSXYZnrc) simulation results showing relative dose values around the interface of two phantom slabs when different configurations of material placements were used: top and bottom phantoms slabs are solid water (SW–SW), the top slab is molded silicone and the bottom slab is solid water (MS–SW), or the top and bottom slabs are molded silicone (MS–MS). Results are shown using a Co‐60 photon beam with a 1.5 cm top phantom slab thickness (a) and a 5.0 cm top phantom slab thickness (b), as well as for a 6 MV photon beam with a top phantom slab thickness of 1.5 cm (c), and a top phantom slab thickness of 5.0 cm (d). In all cases, the dose is presented relative to the dose at 100 cm source‐to‐axis distance (SAD) for the SW–SW setup at each respective depth and beam energy. The field size and SAD for all simulations were 10 × 10 cm^2^, and 100 cm, respectively, and all simulations yielded values with uncertainties below 0.3%. Dose ratios from film measurements made with silicone E10 and E50 types are also shown for comparison and are labeled as (film‐E10) or (film‐E50), for film measurements in each type of silicone material (E10 or E50)

Figure 3 presents the relative doses obtained through MC (DOSXYZnrc), with data plotted along Co‐60 and 6 MV beams’ central axis direction at various depths in various. The values at 100 cm SAD are the relative doses ($D_{\mathrm{SW}-\mathrm{SW}}^{\mathrm{MS}-\mathrm{SW}}$ and $D_{\mathrm{SW}-\mathrm{SW}}^{\mathrm{MS}-\mathrm{MS}}$) presented in Tables 7 and 8. For all beam energies and depths in phantom material, a visible perturbation is present just beyond 100 cm SAD when an interface of silicone and solid water (MS–SW) is present.

## 4 DISCUSSION

MS composites offer practical advantages for constructing deformable anthropomorphic phantoms.^5^, ^12^, ^13^, ^15^, ^34^ With increased utilization of 3D printing in radiotherapy, MSs are also being used to mold custom patient‐specific radiotherapy bolus out of 3D printed shells.^10^, ^35^ In this paper, we constructed slab phantoms out of two types of commercial silicone composites. The first, referred to as E10, formed a soft and flexible slab, and the second, referred to as E50, formed a harder and more rigid slab. The molding process demonstrated was simple and provided a reproducible setup for conducting IC and film dose measurements at the interface of two slab phantom planes.

Due to their mass density and electron density being similar to water's, E10 and E50 were expected to be suitable for applications in MV photon radiotherapy and dosimetry—where Compton scattering interactions dominate. Since film has negligible radiation fluence perturbation effects, and if we consider measurements conducted with Co‐60 and 6 MV photon beams using film as being more reliable than the Markus IC measurements, we can conclude that the relative dose ratios resulting from MS–MS or MS–SW setups were up to 5% different than with a SW–SW setup see (Tables 7 and 8). As an example, for a prescription dose of 200 cGy, this would translate to a delivered dose of 190 cGy at the same depth. In these cases, the differences in dose ratios were more prominent when the phantom setup configuration comprised an interface of two media (MS–SW), as opposed to being fully made of silicone (MS–MS). Indeed, when silicone was used alone (MS–MS) the dose ratios were up to 4% higher and 2% lower in Co‐60 and 6 MV photon beams, respectively (or 208 cGy and 198 cGy, respectively in our stated example above). That is to say that using a phantom made purely of silicone would have more dosimetric tissue equivalence in the higher energy photon beam at measurement depths of 1.5 cm or 5.0 cm. Furthermore, based on its relative dose attenuation in in 6 MV photon beams, E10 (which is more deformable than E50 and is mechanically similar to human tissue^5^) seems to be better suited for phantom and bolus applications.

The difference between measured dose values obtained in E10 and E50 materials may be related to differences in their chemical composition. As mentioned previously, in addition to the repeating silicone polymers in silicone composites, these materials are manufactured to incorporate small amounts of “filler” material. Filler material types range from carbon, to silica, titanium, or barium sulfate. Due to proprietary information, it was not possible to obtain the exact formulation of E10 and E50 from the manufacturer, so the measured dose differences between the two materials could not be identified with certainty to result from differences in filler materials. Only a detailed chemical analysis could offer quantifiable data; however, it is important to note that different silicone composite product lines or different manufacturers can rely on different types and quantities of filler materials to generate variable degrees of hardness or softness, radiopacity, or viscosities for example. Consequently, due to the predominance of the photoelectrical effect at low photon energies, it was expected that the potential presence of higher atomic number elements in E50, which is inferred from its higher shore‐hardness compared to E10, would reflect increased dose attenuation when measurements were conducted in the lower photon energy. Indeed, we found that compared with measurements in SW, using E10 and E50 in 6 MV photon beams caused less dose differences than in Co‐60 photon beams. Dose discrepancies would likely be even more noticeable for kV photon energy ranges, particularly due to the presence of a high amount of silicon (*Z *= 14) in silicone composites (see Table 2).

For all photon beam measurements, dose ratios obtained with the IC were lower than those obtained with film (see Tables 7 and 8). MC simulations were employed to investigate these differences. Simulation results showed that for a generic form of silicone, having no distinction between E10 and E50 at both depths in Co‐60 (see Table 7) and 6 MV photon beams (see Table 8), $D_{\mathrm{SW}-\mathrm{SW}}^{\mathrm{MS}-\mathrm{SW}}$ and $D_{\mathrm{SW}-\mathrm{SW}}^{\mathrm{MS}-\mathrm{MS}}$ dose ratio values had the same trend as those obtained with film.

MC simulations showed that $D_{\mathrm{SW}-\mathrm{SW}}^{\mathrm{MS}-\mathrm{SW}}$ and $D_{\mathrm{SW}-\mathrm{SW}}^{\mathrm{MS}-\mathrm{MS}}\;$values differed at both depths in 6 MV photon beams (see Figure 3). These results resembled those obtained with film measurements. Which again allude to the fact that conducting dose measurements entirely within silicone material will yield results within 2% of those conducted in SW, but larger differences can be expected if silicone is placed on top of SW to create an interface of the two materials. These results can also be clearly visualized from MC simulation data shown in Figure 3, where, at both 1.5 cm and 5.0 cm depths, a reduction in the scored dose is observed at (∼2%) and 0.1 cm beyond (∼4%) the interface of MS–SW phantom configurations. This finding is relevant to consider in applications where silicone composites may be used to mold a bolus for a patient's radiotherapy treatment,^11^, ^36^ or when they are used to construct phantoms for radiotherapy applications using multiple materials.^12^, ^13^, ^15^, ^37^

TPS calculations were also performed using a generic form of silicone. In this case, a corrected RED value of 0.983 was used instead of the TPS determined value of 1.055 ± 0.003. Two dose calculation algorithms (TPS‐CC and TPS‐MC) were applied to establish any potential errors caused by using an algorithm that did not fully account for lateral scatter, such as CC.^38^ In the simple geometry used, no observable differences were found when comparing the point dose ratios obtained with the two dose calculation algorithms (refer to Table 8), where $D_{\mathrm{SW}-\mathrm{SW}}^{\mathrm{MS}-\mathrm{SW}}$ and $D_{\mathrm{SW}-\mathrm{SW}}^{\mathrm{MS}-\mathrm{MS}}$ were all below 1.0%. CC algorithms are generally regarded as accurate for simple geometries,^39^ such as the configurations tested. For accurate TPS dose calculation, and in the case that more complicated calculation geometries and material configuration are to be used, the TPS–MC dose calculation algorithm would offer more reliable results. When an interface of MS–SW was used, results from MC simulations were approximately 2% lower than those from TPS calculations. This is related to the differences in ${\left(\bar{\frac{\mu }{\rho }}\right)}_{\mathrm{water}}^{\mathrm{silicone}}$ and $\;{\left(\bar{\frac{L}{\rho }}\right)}_{\mathrm{water}}^{\mathrm{silicone}}$ (see Table 3), because the TPS will not accurately model silicone's true dose absorption compared to water. During MV photon dose calculation, the Monaco TPS uses the RED value to determine the associated mass density, which, according to Monaco's TPS Dose Calculation Manual,^24^ for silicone's RED of 0.983 equates to a mass density of 0.983 g/cm^3^. Moreover, in the Monaco TPS, the relative mass collisional stopping power for a medium, ${\left(\frac{S_{\mathrm{col}}}{\rho }\right)}_{w\mathrm{ater}}^{\mathrm{med}}$, is calculated as a function of mass density using equations applicable over variable ranges of mass densities, where ${\left(\frac{S_{\mathrm{col}}}{\rho }\right)}_{\mathrm{water}}^{\mathrm{med}}$ = 1.000 between the range of 0.98 < ρ < 1.02.^24^ This is not entirely accurate since, as determined previously,^5^ ${\left(\frac{S_{\mathrm{col}}}{\rho }\right)}_{\mathrm{water}}^{\mathrm{silicone}}$ is 0.948 for 6 MV photon beams. Silicone has a mean excitation energy that is ∼25% higher than water's (see data provided in Table 3) which in turn lowers the mass stopping power for silicone relative to water. This signifies that the TPS does not account for changes in electron fluence when using a medium that is dissimilar to water, and that for accurate TPS‐dose calculation to measurement comparisons, it is necessary to apply a correction to the TPS‐determined dose value. The primary correction to the TPS dose calculation would account for changes in the charge particle fluence which would be reflected by the difference between the TPS applied ${\left(\frac{S_{\mathrm{col}}}{\rho }\right)}_{\mathrm{water}}^{\mathrm{silicone}}$ and its actual value. These corrections alone can be in the order of an increase in calculated dose by 1%–2%.

Contrary to photon measurements, IC and film readings agreed well in 6 MeV electron measurements (refer to Table 9). Here, film results showed the overall differences in dose ratios were within −2% to +4%, depending on the silicone type and setup. TPS calculations showed large discrepancies (> 7%) for $D_{\mathrm{SW}-\mathrm{SW}}^{\mathrm{MS}-\mathrm{MS}}$ with 6 MeV electron beams. Overall, using silicone composites for dose measurements in high‐energy electron beams can be advantageous if the differences between $D_{\mathrm{SW}-\mathrm{SW}}^{\mathrm{MS}-\mathrm{SW}}\;$and $D_{\mathrm{SW}-\mathrm{SW}}^{\mathrm{MS}-\mathrm{MS}}$ are taken into consideration. It is also worth noting that the reported dose ratios in electron beams are high (as opposed to in MV photon beams, where the reported ratios were generally low), meaning that dose measurements in silicone result in a higher dose value than in SW. This can also be attributed to the fact that the collisional mass stopping power for silicone is lower than that of water, and so the magnitude of electron fluence attenuated by silicone would be less than that in water of equal physical thickness. Once again, this finding is relevant to consider in applications where silicone composites may be used to mold a bolus to increase skin dose for a patient's radiotherapy treatment.

The choice of using the Markus IC was based on practicality and offered a conceptualized benefit for establishing the dose readings at the interface of silicone and SW. It would have been challenging to use a Farmer type IC in this type of phantom slab geometry due to the IC slot molding process (see Figure 1) and the larger volume of Farmer IC's which would lead to volume‐averaging effects. Nonetheless, it was found that the Markus IC generally yielded lower readings than with film, and, if these measurements were not corroborated by other tools, would have indicated that $D_{\mathrm{SW}-\mathrm{SW}\;}^{\mathrm{MS}-\mathrm{SW}}$and $D_{\mathrm{SW}-\mathrm{SW}}^{\mathrm{MS}-\mathrm{MS}}$ yielded similar results. IC dose ratios did not significantly differ when the protection cap was used or removed. For 6 MV beams, the range of differences in dose ratios was −0.28% to 1.05% at depths of 1.5 cm and 5.0 cm, respectively. And for 6 MeV beams, the range was −1.14% to −1.37% at a depth of 1.5 cm. The IC readings were found to differ from data obtained by film measurements, TPS calculations, and MC simulations. These inconsistencies are related to how parallel plate ICs are constructed. The Advanced Markus IC is manufactured for absolute dosimetry in high‐energy electron beams and is made of poly‐methyl methacrylate (PMMA) with a 0.03 mm thick polyethylene CH_2_ entrance foil (2.76 mg/cm^2^). Its protection cap is also made of PMMA (0.87 mm thickness and 1.19 g/cm^3^) and has a small sensitive volume with a radius of 2.5 mm (for a depth of 1.0 mm).^40^ Based on these specifications, it is designed to minimize dose perturbation effects and minimize volume averaging in the depth direction, which was necessary for the measurements conducted in this study. This has been previously validated both experimentally and through MC simulations in Co‐60 photon beams, and have shown that the associated correction for attenuation and scatter in the chamber wall (*P*_wall_) is close to unity.^40^, ^41^ Nevertheless, an under‐response in measured dose was still observed in our experimental results for photon beams, in which no measurable difference in relative dose was found between interfaces made by SW−SW and MS−SW. This is due to the fact that the backplate of the parallel‐plate IC is sufficiently thick to be the primary source of backscatter fluence measured by its body.^42^, ^43^, ^44^ This effect may be reduced using a parallel‐plate IC which is more robust to backscatter such as the Roos® (PTW‐Freiburg, Germany). Based on our data, for pre‐clinical dose verification, radiochromic film offers a more reliable alternative for measuring dose in silicone material, as well as different material interfaces, in setups similar to those applied in our study.

This work investigated the use of silicone in open photon and electron beams only, whereas more modulated radiation beams are often encountered in clinical settings. With intensity modulated beams, the use of multi‐leaf collimators can result in low‐energy scatter, which, due to silicone's higher *Z*_eff_, results in a dramatic increase in photoelectric interactions. In these situations, it may be interesting to also evaluate how dose distributions measured in silicone composite materials differ from those measured in SW.

## 5 CONCLUSIONS

MS composites offer practical advantages for constructing customized patient bolus and radiotherapy phantoms for use in high‐energy photon and electron beams. Silicone compositions differ from SW's, and it is important to consider associated differences in beam attenuation properties prior to clinical use or phantom applications. This study demonstrated how the dosimetric properties and effects of silicone can be assessed. Experimental, TPS calculations, and MC simulation data showed that compared with the dose measured in SW, when silicone is used in conjunction with SW to form an interface of two materials, differences in measured dose become relatively high. Using silicone alone offers a more tissue‐equivalent medium for constructing phantoms for use in absorbed dose measurement under high‐energy photon and electron beams.

## AUTHORS’ CONTRIBUTION

Ghada Aldosary and Jason Belec conceived the presented idea. Ghada Aldosary, Jason Belec, Claire Foottit, and Eric Vandervoort planned and carried out the experiments. Ghada Aldosary and Jason Belec developed the theory. Ghada Aldosary conducted the MC simulations, performed the treatment planning system calculations, designed the figures, and drafted the manuscript. All authors discussed the results and commented on the manuscript.

## CONFLICT OF INTEREST

The authors declare that there is no conflict of interest that could be perceived as prejudicing the impartiality of the research reported.
